# Patterns of Circulating Inflammatory Biomarkers in Older Persons with Varying Levels of Physical Performance: A Partial Least Squares-Discriminant Analysis Approach

**DOI:** 10.3389/fmed.2014.00027

**Published:** 2014-09-01

**Authors:** Emanuele Marzetti, Francesco Landi, Federico Marini, Matteo Cesari, Thomas W. Buford, Todd M. Manini, Graziano Onder, Marco Pahor, Roberto Bernabei, Christiaan Leeuwenburgh, Riccardo Calvani

**Affiliations:** ^1^Department of Geriatrics, Neurosciences and Orthopedics, Catholic University of the Sacred Heart, Rome, Italy; ^2^Department of Chemistry, Sapienza University of Rome, Rome, Italy; ^3^Gérontopôle, Centre Hospitalier Universitaire de Toulouse, Toulouse, France; ^4^INSERM UMR1027, Université de Toulouse III Paul Sabatier, Toulouse, France; ^5^Department of Aging and Geriatric Research, University of Florida, Gainesville, FL, USA

**Keywords:** aging, gait speed, inflammaging, cytokines, disability, interleukin, immune senescence, multiplex assay

## Abstract

**Background:** Chronic, low-grade inflammation and declining physical function are hallmarks of the aging process. However, previous attempts to correlate individual inflammatory biomarkers with physical performance in older people have produced mixed results. Given the complexity of the inflammatory response, the simultaneous analysis of an array of inflammatory mediators may provide more insights into the relationship between inflammation and age-related physical function decline. This study was designed to explore the association between a panel of inflammatory markers and physical performance in older adults through a multivariate statistical approach.

**Methods:** Community-dwelling older persons were categorized into “normal walkers” (NWs; *n* = 27) or “slow walkers” (SWs; *n* = 11) groups using 0.8 m s^−1^ as the 4-m gait speed cutoff. A panel of 14 circulating inflammatory biomarkers was assayed by multiplex analysis. Partial least squares-discriminant analysis (PLS-DA) was used to identify patterns of inflammatory mediators associated with gait speed categories.

**Results:** The optimal complexity of the PLS-DA model was found to be five latent variables. The proportion of correct classification was 88.9% for NW subjects (74.1% in cross-validation) and 90.9% for SW individuals (81.8% in cross-validation). Discriminant biomarkers in the model were interleukin 8, myeloperoxidase, and tumor necrosis factor alpha (all higher in the SW group), and P-selectin, interferon gamma, and granulocyte–macrophage colony-stimulating factor (all higher in the NW group).

**Conclusion:** Distinct profiles of circulating inflammatory biomarkers characterize older subjects with different levels of physical performance. The dissection of these patterns may provide novel insights into the role played by inflammation in the disabling cascade and possible new targets for interventions.

## Introduction

Among the host of functional and structural changes entailed by the aging process, physical function decline and chronic, low-grade inflammation represent pervasive features (1, 2).

Over the last decades, there has been increasing recognition of the importance of physical function assessment in advanced age, both as a central component of clinical evaluation and a specific outcome for interventions (3). Walk speed at usual pace over 4 m, hereby referred to as gait speed, is an inexpensive, objective, and easy to interpret test to assess physical performance in the elderly (4). Slow gait speed has indeed been associated with clinical and subclinical conditions (5, 6), and is able to predict several health-related events apparently extraneous to physical function (e.g., incident cognitive impairment, hospitalization, surgical complications, institutionalization, and mortality) (7–10). As such, gait speed is advocated as an additional “vital sign” to be included in the routine clinical assessment of geriatric patients (4, 11).

The chronic, low-grade inflammatory status that accompanies aging results from an imbalance between pro- and anti-inflammatory networks, in the absence of overt infections (“sterile” inflammation) (2). This phenomenon, also termed “inflammaging,” explains several traits of the aging phenotype and is a major risk factor for morbidity and mortality (12). Indeed, inflammaging entails several cytokines, molecular pathways, effector cells, and tissue responses that are shared across a multitude of age-related conditions (13).

Specific circulating inflammatory markers have been associated with adverse health outcomes in older persons. For instance, elevated serum levels of interleukin (IL) 6, tumor necrosis factor alpha (TNF-α), and C-reactive protein (CRP) have been linked with poor function and mobility status (14–18). This evidence would support the assessment of inflammatory biomarkers in the clinical evaluation of elderly patients. Notwithstanding, measurements of inflammatory mediators have not yet been incorporated into standard clinical practice, partly because there is not a “gold standard” inflammatory measurement that reliably predicts incident adverse outcomes in older adults (19). This, in turn, is due to the fact that the effects of inflammation on health outcomes have been inferred mostly through the analysis of single biomarkers. However, these mediators act in a complex and coordinated network in which their functions may be modified, replaced, or modulated by other cytokines. Yet, there have been only few attempts to develop a comprehensive study of inflammatory markers and aggregate biologically informed measures to maximize their predictive validity (20–23).

In the present study, we hypothesized that specific patterns of circulating inflammatory markers would characterize elderly individuals with varying levels of physical performance. To address this research question, an array of inflammatory mediators was assayed simultaneously in blood samples of community-living older adults. Multivariate statistical models were constructed to explore the relationship between systemic inflammatory profiles and physical function.

## Materials and Methods

### Participants

Participants were community-dwelling men and women aged 70 years or older. Recruitment of participants was coordinated by the Recruitment Core of the University of Florida Claude D. Pepper Older Americans Independence Center, as detailed elsewhere (24–26). Recruitment strategies included media articles, direct mailings, newspaper announcements, and presentations to community groups.

A set of eligibility criteria was chosen to minimize the possible confounding effect of comorbid conditions, medications, or lifestyle habits on the relationship between inflammatory profiles and physical performance. Briefly, subjects were not included if presenting with any of the following characteristics: smoking in prior 12 months; engagement in regular physical exercise; history of drug or alcohol abuse; active treatment for cancer or cancer in the past three years; heart failure New York Heart Association class III–IV; stroke with upper and/or lower extremity involvement; Parkinson’s disease or other neurological disorders likely to interfere with physical function; major psychiatric illnesses; peripheral vascular disease Lériche–Fontaine stage 3–4; history of life-threatening cardiac arrhythmias; cognitive impairment (i.e., Mini Mental State Examination score ≤21); renal disease requiring dialysis; lung disease requiring steroids; chronic viral diseases (e.g., hepatitis B and C, HIV); lower extremity amputation; severe knee, or hip osteoarthritis limiting mobility; diabetes with visual, vascular, or neuropathic complications; inflammatory diseases (e.g., rheumatoid arthritis, vasculitis, autoimmune disorders, and inflammatory bowel disease); taking growth hormone, estrogen replacement, testosterone, anticoagulants, steroids, non-steroidal anti-inflammatory drugs on a regular basis; severe obesity [i.e., body mass index (BMI) ≥35]; underweight (i.e., BMI ≤18.5); active weight loss >5 kg in prior 3 months; life-threatening illnesses with an estimated life expectancy <1 year. Subjects on statin treatment were asked to refrain from drug administration 1 month prior to blood drawn upon their general practitioner’s approval. Prior to enrollment in the study, all participants provided written informed consent. The study protocol was approved by the University of Florida’s Institutional Review Board.

### Assessment of physical performance

The physical performance status of participants was assessed by measuring gait speed over a 4-m course at the person’s usual pace. The faster of two trials (meters per second) was used for the analysis. Physical performance testing was conducted at the study center by trained personnel not involved in the investigation. As shown by Guralnik et al. (27), gait speed provides an excellent estimate of lower extremity function and possesses a predictive power for incident disability comparable to composite tools, such as the short physical performance battery (SPPB) (28).

For the purpose of the study, subjects were categorized into slow (SW) or normal walker (NW) groups, using 0.8 m s^−1^ as the cutoff. This threshold was selected based on the evidence that older people walking slower than 0.8 m s^−1^ are at especially high risk of experiencing adverse health-related events, including disability, cognitive impairment, morbidity, institutionalization, falls, and mortality (8–10).

### Blood collection and processing

Blood samples were obtained in the morning by venipuncture of the median cubital vein after overnight fasting, using commercial collection tubes (BD Medical, Franklin Lakes, NJ, USA). For serum extraction, samples were left at room temperature for 20 min and subsequently centrifuged at 1,000 × *g* for 10 min at 4°C. For plasma separation, samples were collected in EDTA tubes and immediately centrifuged at 1,000 × *g* for 10 min at 4°C. Serum and plasma were aliquoted and stored at −80°C until analyses.

### Multiplexed immunoassay for the quantification of inflammatory markers

A panel of 14 inflammatory markers, growth factors, and vascular adhesion molecules, related to systemic and/or vascular inflammation, was measured via a multiplex, magnetic bead-based immunoassay (MILLIPLEX^®^ map; EMD Millipore, Billerica, MA, USA). Analytes were assayed in the serum by the MILLIPLEX^®^ map High Sensitivity Human Cytokine Kit Multiplex Assay (Cat. # HSCYTMAG-60SK) and in the plasma by the MILLIPLEX^®^ map Human Cardiovascular Disease Magnetic Bead Panel 2 – Cardiovascular Disease Multiplex Assay (Cat. # HCVD2MAG-67K). The choice of the analytes was based on previous evidence of their possible involvement in the aging process and the disabling cascade as well as commercial availability. Assays were performed according to the manufacturer’s instructions, except that samples were run in triplicate. The multiplex immunoassay panels were analyzed on a MILLIPLEX^®^ Analyzer 3.1 xPONENT System (Luminex^®^ 200™) and data analysis performed through the MILLIPLEX^®^ Analyst software. The inter-assay coefficient of variation was <5% for the high sensitivity human cytokine kit multiplex assay and <15% for the cardiovascular disease multiplex assay.

### Statistical analysis

All analyses were performed using in-house routines running under Matlab R2011b environment (The MathWorks, Natick, MA, USA).

#### Descriptive statistics

Differences in demographic, anthropometric, clinical, and functional characteristics according to gait speed categories were assessed via the Mann–Whitney *U* test and χ^2^ test, for continuous and categorical variables, respectively. Both tests were two-sided, with statistical significance set at *p* < 0.05.

#### Partial least squares-discriminant analysis of gait speed categories vs. circulating inflammatory markers

The relationship between gait speed categories and patterns of circulating inflammatory biomarkers was explored by constructing and validating a predictive classification model. The approach chosen for the present study was based on Partial least squares-discriminant analysis (PLS-DA) (29), due to its versatility and ability to deal with highly correlated predictors. Briefly, PLS-DA is a classification method based on the PLS regression algorithm (30); thus, in order to introduce the former, it is necessary to describe the latter.

Designating with *X* the matrix of predictors (inflammatory markers) and *Y* the matrix of responses (gait speed categories), the aim of PLS regression was to find the optimal set of coefficients *B* relating *X* to *Y*, according to:

(1) Y=XB

Partial least squares regression is, therefore, a linear regression model, but differs from the classical multiple linear regression approach in that only the relevant part of the information present in the *X* matrix is used for the prediction of *Y*. Indeed, PLS modeling involves projecting the *X* data (which lie onto a *p*-dimensional space, *p* being the number of measured variables) onto a lower-dimensional subspace of so-called latent variables (LVs). LVs are defined as those directions in space where there is the maximum covariance between *X* and *Y* blocks. The representation of data onto the reduced subspace of LVs is captured by the scores matrix *T*:

(2) T=XR

*R* being the projection matrix. It is then the *T* matrix that is used to predict *Y* according to:

(3) $$Y=TQ^{T}$$

where *Q* is a set of coefficients called *Y* -loadings. By combining *R* and *Q*, the regression coefficients *B* defined in Eq. (1) are obtained:

(4) $$B=RQ^{T}$$

Since the scores matrix is low-dimensional and its columns are orthogonal, PLS allows building regression models also for so-called “ill-conditioned” problems, i.e., those cases in which the number of measured variables is larger than the number of individuals and/or the measured variables are highly correlated with each other.

The PLS-DA algorithm was designed to take advantage of these characteristics and to cope with classification problems, for which other methods, such as linear discriminant analysis, suffer the same drawbacks of classical multiple linear regression approaches. To this purpose, the classification problem has to be stated in terms of regression, which is accomplished by means of a dummy-coded vector *y* that accounts for class-belonging. For each participant, the value of *y* was set to 1 if he/she belonged to the NW group and 0 if he/she belonged to the SW class. A standard PLS model was then calculated between the *X* matrix of experimental variables (inflammatory markers) and the dummy-coded vector codifying for class-belonging. The classification was eventually carried out on the basis of the values of *y* predicted by the algorithm. Since the two categories of gait speed were coded as 0 and 1, if the predicted *y* for an individual was greater than 0.5, he/she would be classified as NW. Conversely, for predicted *y* values lower than this threshold, he/she would be classified as SW.

Once the model was calculated, information about the experimental variables more important in the discrimination was obtained by inspecting the so-called variable importance in projection (VIP) indices (31). VIP scores indicate the contribution of each of the measured variables to the PLS model and are scaled so that a “greater than 1” rule can be used to assess statistical significance.

#### Validation of the PLS-DA model

For any statistical model to be reliable, the calibration stage should always be accompanied by a careful validation of its results. The choice of proper validation becomes even more relevant when the phase of model building involves the selection of the optimal value of some adjustable parameters (as it occurs in the case of PLS-DA, where the number of LVs has to be set). Since the optimal values of the parameters are typically chosen as the ones that minimize some kind of error measurement in a dataset (possibly treated as unknowns by the model), this same set of data cannot be used also to evaluate the final model performance (as it would lead to overoptimistic results). When many samples are available, this translates into the necessity of having three sets of data: the calibration set for model building, the internal validation set for the selection of model parameters, and the external validation set (or test set) for the final validation of model performance. However, in cases such as the one of the present study, where the number of participants would not allow extracting three representative sets, re-sampling strategies may be used. In particular, the so-called double cross-validation strategy was adopted (32). Double cross-validation operates by randomly extracting from the available number of samples a small subset to constitute an external validation set. The remaining samples are then divided into a certain number of cancelation groups, like in standard cross-validation, to estimate the optimal model complexity. The optimal model on this subset of data is subsequently validated on the external validation subset. The whole procedure is repeated a suitable number of iterations to obtain a good representativeness and the final results are averaged.

In order to rule out any possibility of chance correlation, the average results obtained from the double cross-validation procedure were further compared with the results of permutation tests. These tests are used to obtain an empirical distribution of the classification figures of merit under the null hypothesis (i.e., under the assumption that no discrimination exists between the two classes), and are carried out by repeating the whole modeling stage on datasets for which the class labels are randomly permuted. In the present study, permutation tests involved 1,000 randomizations. Three figures of merit were considered: (1) the number of misclassifications (NMC), (2) the area under the receiver operating characteristic (ROC) curve (AUROC), and (3) the value of the discriminant *Q*^2^ (*DQ*^2^) (33). NMC is the most intuitive of all diagnostic statistics as it simply indicates the number of samples (or participants, as in the present investigation), which are wrongly classified by the model. AUROC is a figure of merit borrowed from signal processing and is particularly useful to characterize binary classifiers. Its values range between 1 (perfect classification) and 0 (no discrimination). *DQ*^2^ was introduced by Westerhuis et al. (34) as a modification of the standard *Q*^2^ (*R*^2^ in cross-validation) to cope with the peculiarities of classification problems addressed by regression methods. Like its regression analog, *DQ*^2^ assumes its highest values in the case of a perfect discrimination between classes. Differently from standard *Q*^2^, *DQ*^2^ can also be negative (i.e., it is not bound to the 0–1 range of values).

## Results

### Descriptive characteristics of the study sample

A total of 38 community-dwelling older adults were recruited for the study, 27 NWs and 11 SWs. Demographic, anthropometric, functional, and clinical characteristics of participants according to gait speed categories are shown in Table 1. SW subjects were significantly older than NWs (*p* = 0.0118). The average gait speed was 0.62 m s^−1^ (±0.08 SD) in the SW group and 1.10 m s^−1^ (±0.18 SD) among NW participants (*p* < 0.0001). No differences between groups were observed with regard to gender or ethnicity distribution, BMI, and number of comorbid conditions or medications. The average concentrations of circulating inflammatory markers in the two gait speed groups are reported in Table 2.

**Table 1:** **Descriptive characteristics of the study population according to gait speed categories**.

	Gait speed categories		*p*-value
	Normal walkers (*n* = 27)	Slow walkers (*n* = 11)	
Age, years (mean ± SD)	76.4 ± 5.5	81.5 ± 4.9	0.0118
Gender (female), *n* (%)	10 (37.0)	5 (45.5)	0.6300
Ethnicity, *n* (%)			
Caucasian	26	11	0.5179
Afro-American	0	0	
Other	1	0	
BMI (mean ± SD)	26.7 ± 3.7	28.2 ± 4.0	0.2952
Number of medications (mean ± SD)	3.0 ± 2.9	4.0 ± 3.0	0.3893
Comorbidities^a^ (mean ± SD)	0.74 ± 0.98	1.27 ± 1.56	0.2122
Gait speed, m s^−1^ (mean ± SD)	1.10 ± 0.18	0.62 ± 0.08	0.0001

*BMI, body mass index; SD, standard deviation*.

*^a^Includes coronary artery disease, hypertension, prior stroke, peripheral vascular disease, osteoarthritis, chronic obstructive pulmonary disease, and diabetes mellitus*.

**Table 2:** **Serum or plasma concentration of inflammatory biomarkers according to gait speed categories**.

	Gait speed categories	
	Normal walkers (*n* = 27)	Slow walkers (*n* = 11)
	mean ± SD	mean ± SD
GM-CSF, pg mL^−1^^a^	1.99 ± 4.09	0.46 ± 0.71
IFN-γ, pg mL^−1^^a^	6.04 ± 12.28	0.90 ± 1.50
IL1β, pg mL^−1^^a^	0.59 ± 1.14	0.13 ± 0.12
IL5, pg mL^−1^^a^	0.92 ± 1.79	0.61 ± 0.97
IL6, pg mL^−1^^a^	2.90 ± 3.78	3.93 ± 4.39
IL8, pg mL^−1^^a^	3.70 ± 1.67	4.34 ± 1.46
IL10, pg mL^−1^^a^	37.00 ± 58.31	27.02 ± 33.79
IL12(p70), pg mL^−1^^a^	4.21 ± 13.49	10.29 ± 31.44
IL13, pg mL^−1^^a^	5.25 ± 10.56	3.90 ± 8.65
TNF-α, pg mL^−1^^a^	7.96 ± 4.58	8.21 ± 2.91
MPO, ng mL^−1^^b^	24.91 ± 13.68	32.80 ± 21.59
P-selectin, ng mL^−1^^b^	53.55 ± 36.52	34.46 ± 14.69
sICAM-1, ng mL^−1^^b^	86.67 ± 86.41	63.06 ± 19.27
sVCAM-1, ng mL^−1^^b^	1088.00 ± 882.80	870.10 ± 116.60

*SD, standard deviation*.

*^a^Serum analyte*.

*^b^Plasma analyte*.

*GM-CSF, granulocyte macrophage colony-stimulating factor; IFN-γ, interferon gamma; IL, interleukin; MPO, myeloperoxidase; sICAM-1, soluble intercellular adhesion molecule; sVCAM-1, soluble vascular cell adhesion molecule 1; TNF-α, tumor necrosis factor alpha*.

### Participant classification according to PLS-DA

Given the different age distribution of participants belonging to the two gait speed categories, a preliminary analysis was conducted to rule out the existence of a relationship between inflammatory markers and age. To this purpose, a PLS model relating these variables was built and validated. The optimal model, which included five LVs and accounted for 82.8% of the *X* and 46.8% of the *Y* variance, showed very poor performance both in calibration [Root Mean Squared Error (RMSE) = 4.2; *R*^2^ = 0.48] and in sixfold cross-validation [RMSE cross-validation (RMSECV) = 6.2; *Q*^2^ = 0.01]. This observation indicates that, in our study sample, circulating levels of inflammatory mediators are not significantly influenced by age.

**Figure 1:**
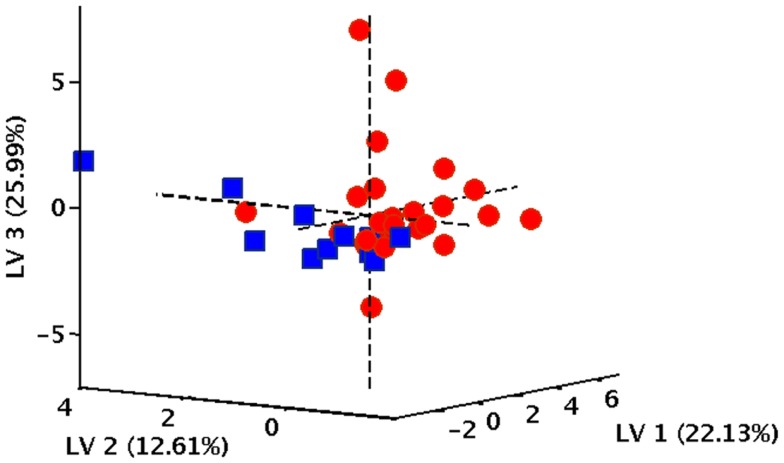
**Projection of participants onto the space spanned by the first three latent variables (LVs) of the PLS-DA model**. Red circles correspond to normal walkers, blue squares identify slow walkers.

In order to verify the existence of specific patterns of inflammatory markers in participants with varying gait speed performance, a PLS-DA classification model was constructed and validated. The optimal PLS-DA model was built using five LVs that accounted for more than 75.4% of the variance originally present in the *X* block. As indicated by the double cross-validation procedure, the model allowed to correctly predict the gait speed category in 89.9% of participants in the calibration phase (90.9% for NWs and 88.9% for SWs), 77.9% in the internal validation stage (81.1% for NWs and 74.1% for SWs), and 71.1% in the external validation (73.7% for NWs and 69.8% for SWs). The classification ability of the model is also evident by inspecting the projection of participants onto the space spanned by the first three LVs of the PLS-DA model (Figure 1), which shows a clear separation between subjects assigned to the two gait speed groups.

**Figure 2:**
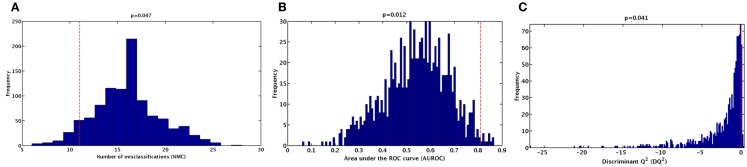
**Distribution of (A) number of misclassifications (NMC), (B) area under the ROC curve (AUROC), (C) and discriminant *Q2* (*DQ2*) values under their respective null hypothesis as estimated by permutation tests with 1,000 randomization (blue histograms) and corresponding values obtained by the PLS-DA model on unpermuted data (red dashed lines)**.

To further validate the classification model and rule out the possibility of chance correlations, the results of the double cross-validation procedure were compared with the distributions of specific figures of merit under the null hypothesis. The distribution of NMC, AUROC, and *DQ*^2^ under their respective null hypothesis, as estimated by the permutation tests, is reported in Figure 2. The corresponding values obtained by the PLS-DA model on unpermuted data, as evaluated by the double cross-validation procedure, are also shown. The results of the PLS-DA classification model were statistically significant, as for all of the three figures of merit, *p-*values lower than 0.05 were obtained.

In order to identify the inflammatory markers, which were mostly involved in discriminating between gait speed categories, the values of the VIP indices were inspected. The variables corresponding to a VIP greater than 1 are reported in Table 3. Six inflammatory markers were found to contribute significantly to the discrimination model. The sign of the regression coefficients of the PLS-DA model is reported to indicate how the concentration of each analyte varies between SW and NW groups. Since the latter was coded as +1 in the dummy *y* vector, all biomarkers with positive regression coefficient had a higher concentration in NWs relative to SWs, and *vice versa*. Accordingly, SWs were characterized by higher circulating levels of IL8, myeloperoxidase (MPO), and TNF-α, and lower levels of P-selectin, interferon gamma (IFN-γ), and granulocyte macrophage colony-stimulating factor (GM-CSF).

**Table 3:** **Inflammatory biomarkers mostly involved in the discrimination between gait speed categories**.

Inflammatory marker	VIP	Sign of regression coefficient
P-selectin	3.37	+
IL8	1.73	−
IFN-γ	1.20	+
MPO	1.14	−
TNF-α	1.07	−
GM-CSF	1.04	+

*VIP, variable importance in projection; GM-CSF, granulocyte macrophage colony-stimulating factor; IFN-γ, interferon gamma; IL8, interleukin 8; MPO, myeloperoxidase; TNF-α, tumor necrosis factor alpha*.

## Discussion

A large number of studies have explored the relationship between inflammation and physical performance in older persons. Apart from few exceptions, most reports relied on single mediators as markers of the inflammatory state, with CRP, IL6, and TNF-α often playing the role of the “usual suspects” (14–18, 35, 36). However, the inflammatory system is highly complex and involves several cellular components of the innate (i.e., neutrophils, macrophages, and mast cells) and adaptive (i.e., B and T lymphocytes) immune response. The inflammatory network is modulated by a host of mediators, such as chemokines, pro- and anti-inflammatory cytokines, growth, and angiogenetic factors, and metabolic markers (e.g., adipokines), under reciprocal control through multiple feedback mechanisms (37). Thus, focusing on single inflammatory mediators may not be sufficient at capturing the intimate relation linking inflammation and physical function in advanced age. This complexity may also help explain the conflicting results reported in the vast literature covering the topic (19).

In an attempt to address this issue, we built and validated a predictive classification model based on PLS-DA to characterize the patterns of circulating inflammatory mediators associated with different levels of physical performance. Our analysis unveiled that older persons with a gait speed above the critical threshold of 0.8 m s^−1^ were characterized by higher circulating levels of P-selectin, IFN-γ, and GM-CSF. Conversely, higher levels of IL8, MPO, and TNF-α defined the inflammatory profile of participants walking slower than 0.8 m s^−1^.

Only sparse reports exist that assessed arrays of cytokines in aging populations to determine, which combination of measures were best at predicting mortality, functional decay, or other adverse health-related events (20–23). Using data from the InCHIANTI (Invecchiare in Chianti; Aging in the Chianti Area) study, Bandeen-Roche et al. (20) developed an index based on seven circulating inflammatory markers (CRP, IL1β, IL1 receptor antagonist, IL6, IL18, transforming growth factor beta 1, and TNF-α), which was independently associated with worsening mobility function and frailty risk. In the Health, Aging, and Body Composition study, a multivariate statistical analysis based on principal component analysis was conducted to identify the “best” combination of inflammatory markers associated with physical function (21). Two principal components were identified, one related to TNF-α and the other to CRP, which were associated with specific physical function measures. In agreement with these reports, our findings support the idea that distinct patterns of variation in inflammatory markers, rather than a generalized elevation of single pro-inflammatory mediators, are associated with functional outcomes in older adults.

Although our study was not designed to address mechanistic hypotheses, it can be speculated that the inflammatory profiles characterizing NW and SW participants may reflect particular aspects associated with innate immune system senescence. IL8 is a chemokine that targets polymorphonucleates (PMNs) and promotes their adhesion, degranulation, respiratory burst, and the synthesis of eicosanoids (38). TNF-α stimulates phagocytosis, degranulation, and oxidative burst activity of PMNs, as well as their migration through the endothelium via up-regulation of endothelial adhesion molecules (39). Remarkably, IL8 and TNF-α regulate the generation of each other (40, 41). Furthermore, the exposure of neutrophils from healthy subjects to TNF-α and/or IL8 enhanced free radical production through the activation of NADPH oxidase and MPO (42).

The finding of higher circulating levels of IFN-γ and GM-CSF in NW participants is in apparent contrast with the role traditionally attributed to inflammation as a contributing factor to the disabling process. However, recent evidence indicates that the capacity to produce IFN-γ and GM-CSF upon stimulation is positively associated with muscle mass and handgrip strength in older men (43). Hence, the preservation of specific inflammatory properties may protect against physical function deterioration in late life, perhaps by acting in a hormetic-like fashion.

Further to this point, P-selectin emerged as the most important inflammatory mediator in the discrimination between gait speed categories, with higher levels found in NW elderly. Selectins are adhesion molecules that mediate the initial rolling of leukocytes along endothelial cells and activated platelets before their firm adhesion and diapedesis at sites of tissue injury and inflammation (44). Increased serum levels of P-selectin have been observed in various cardiovascular disorders, indicating that this biomolecule may serve as a marker for atherosclerosis and endothelial dysfunction (45). Nevertheless, the existence of a complex age-dependent relation between circulating P-selectin levels and cardiovascular disease has been reported (46). Indeed, individuals older than 65 years with documented coronary artery disease showed significantly lower P-selectin levels than did healthy peers (46). Hence, elevated serum levels of P-selectin may be beneficial in some circumstances by preventing the inappropriate activation of neutrophils in the circulation (47). Whether P-selectin exerts protective actions against physical function decline in advanced age, as our data seem to suggest, deserves further investigation.

Although reporting novel findings, our study presents some limitations that need to be acknowledged. First, analyses were conducted in a relatively small group of subjects and involved a vast array of experimental variables. However, the PLS-DA approach is particularly suited for such experimental design because it allows analyzing matrices in which (1) the number of variables is larger than the number of individuals, (2) the variables are correlated with each other, and (3) the differences in biological parameters could be subtle and highly variable among the individuals (34). Moreover, the double cross-validation procedure confirmed the reliability of the PLS-DA model (32, 33). Since the purpose of the investigation was to explore the relationship among aging, inflammation, and functional status, the eligibility criteria were quite restrictive. This approach does not allow extending the results to severely ill, multimorbid subjects. In addition, the study sample was mostly comprised of Caucasian individuals, which impedes generalizing the findings to other ethnic groups. Although only subjects not engaged in regular physical exercise were enrolled, the amount of physical activity of participants was not quantified. Hence, the relationship between inflammatory profiles and the overall level of physical activity could not be established. The absence of longitudinal data on the panel of inflammatory biomarkers obliged us to use a single time-point measurement. No inference on the temporal relationship between changes in inflammatory biomarkers and physical function decline can therefore be drawn. Finally, although a fairly large number of inflammatory biomolecules were assayed, we could obviously not consider all known mediators. Notably, CRP was not measurable with the multiplex assay kits chosen for the present study. Hence, it cannot be excluded that more powerful predictors of physical function might be obtained through the analysis of a larger range of biomediators.

## Concluding Remarks

Findings from the present study indicate that specific patterns of circulating inflammatory markers characterize older persons with different levels of physical performance, as estimated by gait speed. The multivariate analytical strategy adopted allowed overcoming the “one mediator fits all” paradigm (19) and identified robust relationships between clusters of inflammatory biomolecules and physical function levels. This initial investigation could, therefore, pave the way for the identification of novel sets of biomarkers related to physical performance in older individuals. This, in turn, may provide new predictors of disability to be implemented in the clinical arena and possible biological targets for preventive interventions.

## Conflict of Interest Statement

The authors declare that the research was conducted in the absence of any commercial or financial relationships that could be construed as a potential conflict of interest.
